# Local resection of the stomach for gastric cancer

**DOI:** 10.1007/s00595-016-1371-z

**Published:** 2016-06-24

**Authors:** Shinichi Kinami, Hiroshi Funaki, Hideto Fujita, Yasuharu Nakano, Nobuhiko Ueda, Takeo Kosaka

**Affiliations:** Department of Surgical Oncology, Kanazawa Medical University, 1-1 Daigaku, Uchinada-machi, Kahoku-gun, Ishikawa, 920-0293 Japan

**Keywords:** Gastric cancer, Local resection, Laparoscopic endoscopic cooperative surgery, Sentinel node

## Abstract

The local resection of the stomach is an ideal method for preventing postoperative symptoms. There are various procedures for performing local resection, such as the laparoscopic lesion lifting method, non-touch lesion lifting method, endoscopic full-thickness resection, and laparoscopic endoscopic cooperative surgery. After the invention and widespread use of endoscopic submucosal dissection, local resection has become outdated as a curative surgical technique for gastric cancer. Nevertheless, local resection of the stomach in the treatment of gastric cancer in now expected to make a comeback with the clinical use of sentinel node navigation surgery. However, there are many issues associated with local resection for gastric cancer, other than the normal indications. These include gastric deformation, functional impairment, ensuring a safe surgical margin, the possibility of inducing peritoneal dissemination, and the associated increase in the risk of metachronous gastric cancer. In view of these issues, there is a tendency to regard local resection as an investigative treatment, to be applied only in carefully selected cases. The ideal model for local resection of the stomach for gastric cancer would be a combination of endoscopic full-thickness resection of the stomach using an ESD device and hand sutured closure using a laparoscope or a surgical robot, for achieving both oncological safety and preserved functions.

## Introduction

Lymph node dissection in cancer surgery is important as it often provides accurate staging information [1]. In certain cancers, lymph node dissection also plays a role in improving survival outcomes. Gastric cancer with metastasis confined to the regional lymph nodes, has a favorable treatment outcome with gastrectomy involving standard lymph node dissection of an extent up to D2 [2, 3]. Consequently, patients undergoing D2 lymph node dissection for gastric cancer require a wide range of gastrectomy procedures, including either a distal partial gastrectomy or a total gastrectomy. Various postgastrectomy symptoms are known to routinely occur after these procedures [4]. The benefits of jejunal pouch interposition and other such innovative reconstructive approaches, as measures to prevent these symptoms, remain to be definitively proven. An effective countermeasure is to reduce the extent of gastrectomy. Partially reducing the extent of lymph node dissection from D2 would allow for a reduction in the extent of gastric resection. As limited surgeries, the Japanese Gastric Cancer Treatment Guidelines [5] mention pylorus-preserving gastrectomy, which preserves the pylorus and omits the number ‘5’ lymph node dissection along the right gastric artery, and proximal gastrectomy which preserves the distal stomach and omits the dissection of the number ‘4d’, ‘5’, and ‘6’ lymph nodes. Though these procedures alleviate the postoperative symptoms relative to standard surgery, they fail to eliminate them [6, 7]. Local resection confined solely to sites where the gastric cancer is present and omitting lymph node dissection is, therefore, the ideal surgical strategy for preventing postoperative symptoms.

“Local resection of the stomach” refers to a surgical procedure involving full-thickness, local resection of a part of the gastric wall. It is synonymous with “wedge resection”. This term, however, does not apply to resections that include the pylorus or cardia. Annular resection of the stomach along its minor axis is called segmental gastrectomy, and it is not to be confused with local resection.

This paper reviews the indications and procedures for local resection of the stomach, and looks at the future of this procedure for gastric cancer.

## Indications for local resection for gastric cancer: the indications and current concepts

Local resection of the stomach is considered only in the absence of lymph node metastasis, thus eliminating the need for lymph node dissection and resulting in a preserved vascular supply for the stomach. However, because gastric cancer is very common in East Asia, the clinico-pathological basis of lymph node metastasis has been extensively studied, thus allowing for the reliable preoperative identification of gastric cancer cases that are negative for lymph node metastasis.

Centers specializing in the management of gastric cancer have routinely performed local resections of the stomach for early gastric cancers since the end of the twentieth century. While opinions are divided on the indications for local resections of the stomach, there is consensus in its use for small mucosal gastric cancers [8–11]. Table 1 shows the representative indications for local resection of gastric cancers. These indications are based on each individual center’s experience with resections for early gastric cancers in the absence of lymph node metastasis. Laparoscopic local resection of the stomach has also been attempted in centers with laparoscopic proficiency. Ohgami et al. [11] developed a method of lesion lifting that has earned popularity due to its simplicity.

**Table 1:** Old indications for performing local resection available in pertinent Japanese articles

Author	Year	Indications
Kitaoka	1983	(1) Mucosal cancer without ulceration
		(2) Slight submucosal cancer under 20 mm
Ohara	1985	Elevated type cancer under 5 mm
Iwanaga	1989	(1) Mucosal cancer under 5 mm
		(2) Elevated mucosal cancer under 20 mm
Ohgami	1993	(1) Elevated mucosal cancer under 25 mm
		(2) Depressed mucosal cancer under 15 mm

The approach to gastric cancers, however, has changed dramatically with the advent of endoscopic treatment. The first major change followed the development of endoscopic mucosal resection (EMR). EMR is described as a method to resect gastric mucosa using a high-frequency snare [12]. EMR has advantages over surgical local resection of the stomach. First, it does not require the administration of general anesthesia, and thus can also be performed in patients who are otherwise unfit for anesthesia. Second, a successful EMR preserves the entire stomach and such patients thus do not have any postgastrectomy symptoms. However, the technical difficulty in performing EMR and the higher rate of local residual lesions (10–35 %) are its main disadvantages [13]. Many surgeons, therefore, considered EMR as an alternative rather than standard treatment, resulting in inconsistencies in management strategies and a lack of clarity in the choice between EMR and laparoscopic local resection of the stomach in any given case. The second important change was the emergence of endoscopic submucosal dissection (ESD) [13]. Developed by Hosokawa et al. as an improved method for EMR, ESD involved the use of a high-frequency device, inserted through the forceps channel of a gastroscope, to create a circumferential mucosal incision with wide margins followed by submucosal dissection, and thereby achieve en bloc resection of the lesion. Unlike EMR, ESD offers reliable enbloc resection with wide margins, thus being associated with the advantages of an accurate pathological diagnosis and a low rate of residual disease. It is indicated in lesions where the likelihood of being positive for lymph node metastasis is below the operative mortality rate, based on the experience of two high-volume centers and including 5000 cases of surgical resections [14]. The indications for ESD include: (1) differentiated mucosal cancers free of ulcers, (2) differentiated mucosal cancer 3 cm or smaller, accompanied by ulcer scars, (3) differentiated cancer classified as SM1, which are 3 cm or smaller, and (4) undifferentiated mucosal cancers 2 cm or smaller, free of ulcers. These are broad indications and include all previously reported indications for surgical local resection of the stomach. ESD has gained widespread popularity, offering success in conditions where resections are difficult, and also demonstrating favorable outcomes [15]. Consequently, all early gastric cancers negative for lymph node metastasis are considered for ESD, obviating the need for local resection of the stomach for gastric cancer.

## Present procedures for local resection of the stomach

### Laparoscopic local resection

**Fig. 1:**
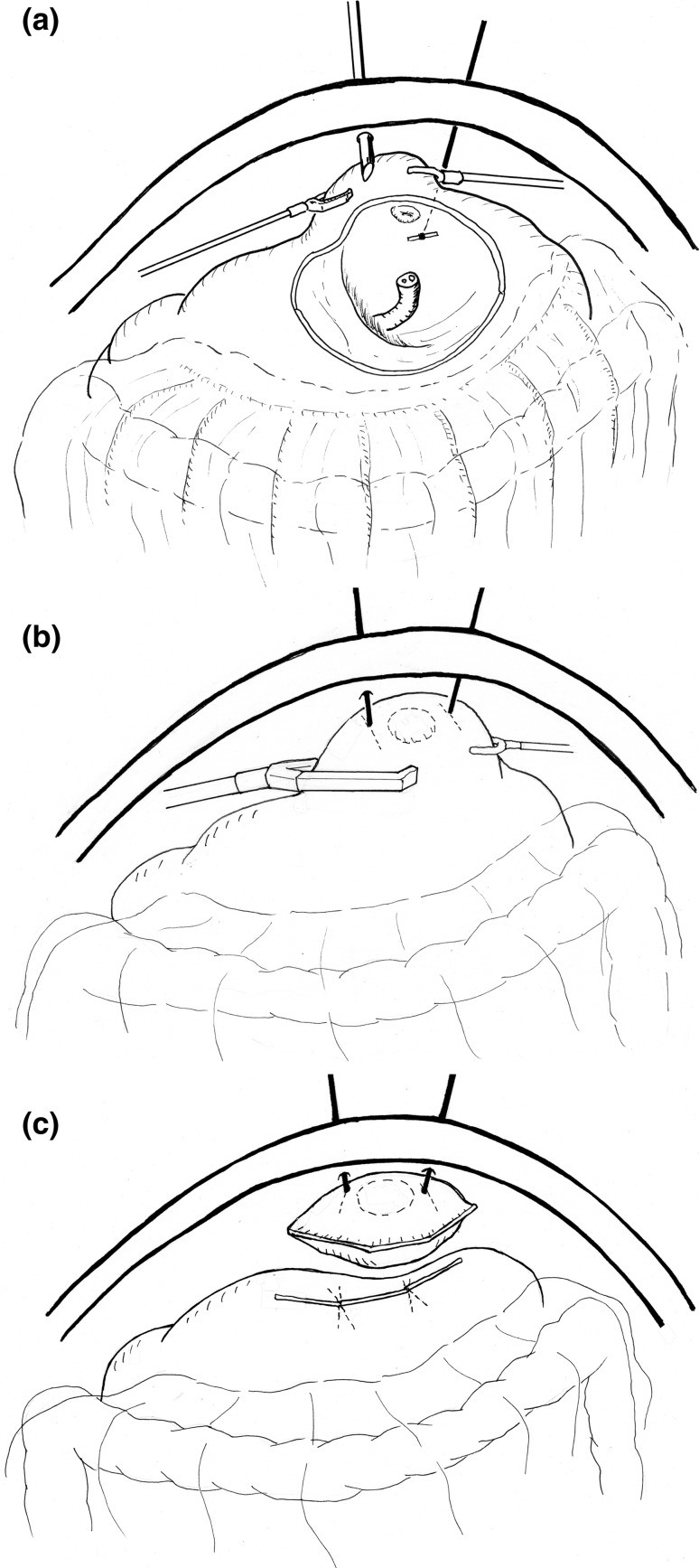
The lesion lifting method for laparoscopic local resection. **a** With the patient in the supine position, under gastroscopic guidance, a gastric puncture is made on the T-bars of a stomach wall lifting device, transabdominally. **b** The lesion is then lifted with the two T-bars, and multiple firings of the linear stapler aid in en bloc resection of the lesion. The entire procedure is performed under gastroscopic vision to ensure that the lesion is not included in the stapled suture line. **c** After performing resection and suturing simultaneously using a linear stapler, the specimen is collected and removed in a specimen collection bag

Laparoscopic local resection has been increasingly performed since the description of the lesion-lifting method by Ohgami et al. [11]. The lesion-lifting method is groundbreaking in that resection of the lesion and stomach wall suturing are performed simultaneously using a linear stapler. Figure 1 summarizes the technique of the lesion-lifting method. This is a simple technique but its application is limited to only small lesions.

**Fig. 2:**
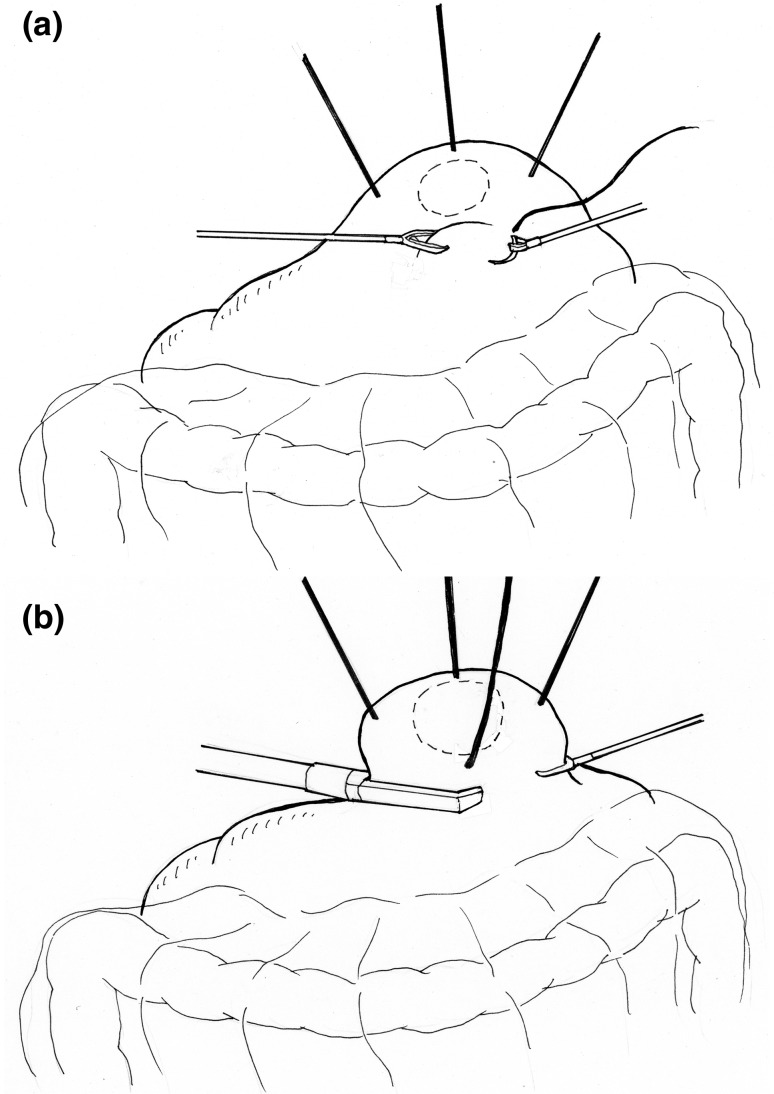
The non-touch lesion lifting method for laparoscopic local resection. **a** The traction sutures placed laparoscopically in the healthy serosa on the periphery of the tumor replace the role played by T-bars. **b** The lesion is lifted using these sutures and en bloc resection is achieved by the multiple firings of a linear stapler

Non-touch lesion lifting [16] is another method described for laparoscopic local resection of the stomach (Fig. 2). This is an excellent method for resecting submucosal tumors, such as GIST, that form a hard mass. However, the possibility of a shear occurring between the serosa and the mucosa renders it difficult to achieve the precise resection of mucosal lesions. This technique could potentially result in a greater than necessary resection of the mucosa or gastric wall, and consequently cause gastric deformation when large lesions are resected.

### Local resection of the stomach using a flexible gastroscope

Endoscopic full-thickness resection (EFTR) is another alternative to laparoscopy, where a gastroscope is used for an intraluminal approach to achieve full-thickness resection of the stomach [17, 19].

Gastric wall perforation is a complication associated with EMR [13] that can follow unintended full-thickness resection when the entire thickness of the stomach wall is mistakenly fastened instead of fastening the submucosal layer during snaring. This is a serious complication but it can be treated without further surgery if the perforation can be closed with endoscopically placed clips. The frequency of perforation is higher with ESD than for EMR, but the techniques for closing perforations have been continually improved, and it is rare for a perforation alone to necessitate a surgical procedure [18]. EFTR refers to a technique of performing a planned full-thickness gastrectomy with a gastroscope, and closing it endoscopically, based on the techniques described for endoscopic closure of stomach perforations. The principal lesions managed by EFTR are small submucosal tumors.

Currently, reports describe EFTR for GIST with small intraluminal growths [20–23]. The significance of EFTR with such lesions is that the extent of the stomach wall that is lost during the procedure is small and laparoscopic surgery can thus be avoided. The drawbacks associated with EFTR are that the tumor size suitable for resection is limited, it is difficult to accurately design the extent of resection, endoscopic stomach wall closure is difficult, often requiring eventual laparoscopic suturing, bleeding can occasionally be challenging, and potential risks of causing injury to other organs exist. For these reasons, EFTR should still be considered as an investigative therapy. However, with the recent introduction of the over-the-scope clip (OTSC), which is a special device for gastric perforation closure, it has become easier than before to close stomach wall defects [24]. There is potential for technical improvements in EFTR and for its indications for use are thus expected to be expanded beyond resection of small GISTs in the future.

### Local resection of the stomach performed using both a laparoscope and a gastroscope

**Fig. 3:**
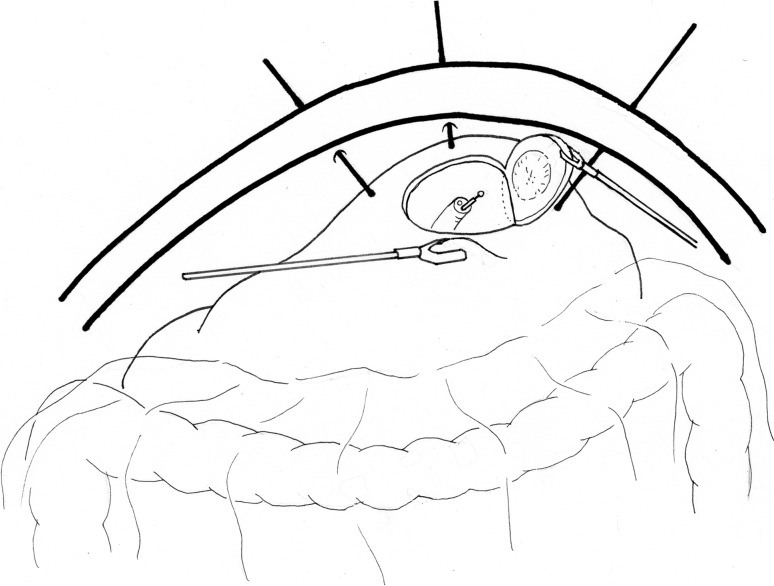
Laparoscopic endoscopic cooperative surgery for local resection. This figure shows the crown method for small cancer. The traction sutures placed laparoscopically in the healthy serosa on the periphery of the tumor. Under the traction of the lesion by these sutures, a full-thickness resection is performed by the endoscopist using the ESD devices. After the lesion is removed intraluminally using the gastroscope, the defect of the stomach wall is closed by hand suturing using a laparoscope

As stated previously, it is difficult to determine the optimal resection line with laparoscopic local resection of the stomach, with the consequence of potential major stomach wall resection resulting in either deformation or impaired emptying. On the other hand, minimal resection is possible with EFTR, but it has problems with stomach wall suture closure, and size and site limitations. The question that arises is whether these limitations can be overcome by simultaneously resecting, with the gastroscope incising the mucosa, and laparoscope incising the serosal layer, thereby enabling safe resection with little deformation. Such techniques have been developed and are collectively referred to as laparoscopic endoscopic cooperative surgery (LECS) (Fig. 3).

The notion of using both a laparoscope and gastric endoscope to perform local resection of the stomach is not new, and laparoscopic–endoscopic collaboration has already been practiced alongside lesion-lifting techniques. The novelty of LECS is that the mucosal incision technique of ESD is first used to determine the resection line from the mucosal side. LECS was first developed by Hiki et al. [25] as an improvement of laparoscopic local resection of the stomach for GISTs measuring 5 cm and smaller. First, a full-circumference mucosal incision is performed by the endoscopist as in ESD. The serosal layer is then incised by the surgeon laparoscopically with the help of an ultrasonic activated device. This incision is deepened to include the remaining thickness of the stomach wall. Before complete excision, the tumor is turned over to the serosal aspect. The final excision is achieved simultaneously with closure of the defect by firing a linear stapler across it. The tumor is collected and removed in a specimen collection bag. Currently, LECS is used as a general term for procedures involving a combination of ESD and laparoscopy for operative procedures involving the gastrointestinal tract [26]. Unlike EFTR, LECS is widely and routinely performed, and is constantly evolving with several researchers reporting various improvements. With classical LECS, the gastric lumen opens into the abdominal cavity with occasional spillage of gastric contents into the peritoneal cavity. Intraperitoneal spillage in the case of gastric cancer, however, is associated with the risk of tumor dissemination. Therefore, applying LECS to early gastric cancer would require a method to prevent the intraperitoneal spillage of gastric contents. The reported methods to prevent spillage, including inverted LECS (Crown method) [27], CLEAN-NET [28], and NEWS [29], have advantages as well as limitations, and none has so far managed to gain widespread popularity.

## Challenges and future prospects of local resection for gastric cancer

As stated above, the advent of ESD and its growing popularity may have ended the role of local resection of the stomach for early gastric cancers. However, whether this has relegated local resection of the stomach as an operation of the past or not has to be discussed.

### Challenges and future prospects regarding indications

Currently, in Japan, while most differentiated mucosal cancers are resected by ESD, surgery is reserved for submucosal cancers and poorly differentiated adenocarcinoma. Surgery, in most of these cases involves laparoscopic gastrectomy [30] with lymph node dissection up to ‘D1+’. Among these cases of surgical resections, only 20 % have lymph node metastasis. This translates to mean that nearly 80 % of such cases have undergone an unnecessary lymph node dissection and as a result, the extent of their gastrectomy is excessive. Better techniques to preoperatively identify the presence or absence of lymph node metastasis accurately would increase the proportion of early gastric cancer cases avoiding unnecessary dissection, and restore the importance of local resections of the stomach.

There is, however, no method other than a conventional clinico-pathological analysis to identify cases that are negative for lymph node metastasis preoperatively. The image-based diagnosis of lymph node metastasis in gastric cancer thus still remains inadequate. This is because most lymph node metastases in early gastric cancer are microscopic, and they may be observed even in very small lymph nodes [31]. The most accurate method of identifying lymph node metastasis currently is intraoperative diagnosis by means of a sentinel lymph node (SLN) biopsy. The sentinel nodes are lymph nodes that are the first to receive the lymph flow from the cancer foci [32]. The Sentinel node concept states that the first sign of lymph node metastasis of cancer is micrometastasis to the sentinel nodes implying negative status for lymph node metastasis in the absence of metastasis in the sentinel nodes. The validity of the SLN concept for early gastric cancer has been deliberated extensively [33–37] with a recent multicenter prospective study demonstrating the SLN theory to be true [38]. SLN biopsy has a sensitivity of 93 % and accuracy of 99 %, suggesting that SLN biopsy is a reliable indicator to consider before deciding whether or not to perform lymph node dissection.

However, though the validity of SLN biopsy in early gastric cancer has been established, there is considerable variation in its diagnostic accuracy. SLN biopsy requires co-operation between several departments, is technically difficult, and has a steep learning curve, thus making it an advanced diagnostic technique to master. The use of SLN biopsy as an indicator of early gastric cancer with a lymph node negative status to apply local resection of the stomach would require further innovations in SLN biopsy techniques to overcome the need for technical proficiency. Such a breakthrough is expected from fluorescence-guided SLN biopsy [39, 40] and the development of new tracers [41].

### Challenges and future prospects regarding resection procedures

While SLN biopsy may identify appropriate cases, local resection for early gastric cancer faces different technical hurdles than that for GIST.

First, since an accurate preoperative diagnosis of the invasiveness of gastric cancer on histology is difficult, ensuring negative margins is thus also difficult. In certain cases of early gastric cancer, it is difficult to determine the extent of infiltration if the cancer has occurred in the background of severe atrophic gastritis [42]. Additionally, poorly differentiated adenocarcinoma may present with a spread that is greater than expected due to the coexistence of noncontiguous crypt progression, which is not obvious on endoscopy [43]. In contrast to GIST, where curative resections are achieved with small margins, curative resection for gastric cancer requires an accurate preoperative assessment of the extent to ensure that the recommended margins are maintained. However, if the margins are too wide, then the benefits of local resection are lost.

A second hurdle pertains to preventing intraperitoneal spillage of the gastric contents into the peritoneum. There are reports of recurrence due to peritoneal metastasis in cases of early gastric cancer complicated by perforation occurring during ESD [44]. The incidence of intraperitoneal metastasis is possibly related to tumor spillage accompanying gastric perforation. Therefore, in local resections for gastric cancer, it is necessary to prevent intraperitoneal spillage of the gastric contents and minimize tumor exposure [26]. While this can be achieved with lesion-lifting, it can be difficult to do so with EFTR and LECS. Various techniques for preventing the spillage of gastric contents into the abdominal cavity and completing LECS have been developed [26–29]. A method called sealed LECS has been reported, in which the resection site is sealed from the serosal surface before LECS is performed [45]. However, laparoscopic surgery currently has limitations in developing such techniques, due to the fact that the freedom for operating the instruments and the viewing angles are limited. Robotic surgery [46] may provide an ideal solution to these difficulties.

### Challenges and future prospects for the significance of local resection of the stomach

The reason for choosing local resections for early gastric cancer is to preserve the stomach volume and minimize functional impairment, but the true benefits of local resection of the stomach in terms of postoperative complaints or functional advantages compared to more radical surgery are not clear. Favorable results have been reported in studies on smaller numbers of patients. However, it should be noted that, occasionally, patients suffer from severe postoperative symptoms due to delayed gastric emptying [47]. It is known that gastric motility is adversely affected by lymph node dissection resulting in vagal denervation of the pylorus or stomach. Kinami, after investigating cases of limited surgery requiring subsequent corrective surgery, observed that avoiding functional impairment after local resection requires ensuring an ample blood flow to the gastric remnant and preventing deformation during resection [47].

The disadvantages arising secondary to preservation of a large part of the stomach also cannot be ignored. First is the elevated risk of cancer occurring in the gastric remnant. Metachronous multiple gastric cancer often occurs in the gastric remnant after distal partial gastrectomy [48], and ESD [49]. Therefore, in cases where local resections are performed for gastric cancer, its frequency would probably be higher than the incidence of cancer in the gastric remnant after distal partial gastrectomy. The most important action to consider is to detect metachronous multiple gastric cancer earlier. Regular endoscopic follow-up to discover gastric remnant cancers at a stage amenable to ESD will neither adversely affect prognosis, nor result in loss of stomach volume. The problem to solve is to identify the appropriate interval for endoscopic follow-up. The next important action is prevention. The identification of cases at high risk for recurrence of cancer in the gastric remnant may offer the possibility to consider them for routine surgery or to plan stringent follow-up protocols. High-risk cases include those with synchronous multiple gastric cancers, metachronous multiple gastric cancers after ESD, and those with a family history of gastric cancer. In the future, profiling using molecular biology techniques may make it possible to screen for cases at risk for multiple gastric cancers. Another preventive measure is to eradicate *Helicobacter pylori*, which is currently carried out in cases undergoing ESD [50].

### Local resection of the stomach for gastric cancer: an ideal model

**Fig. 4:**
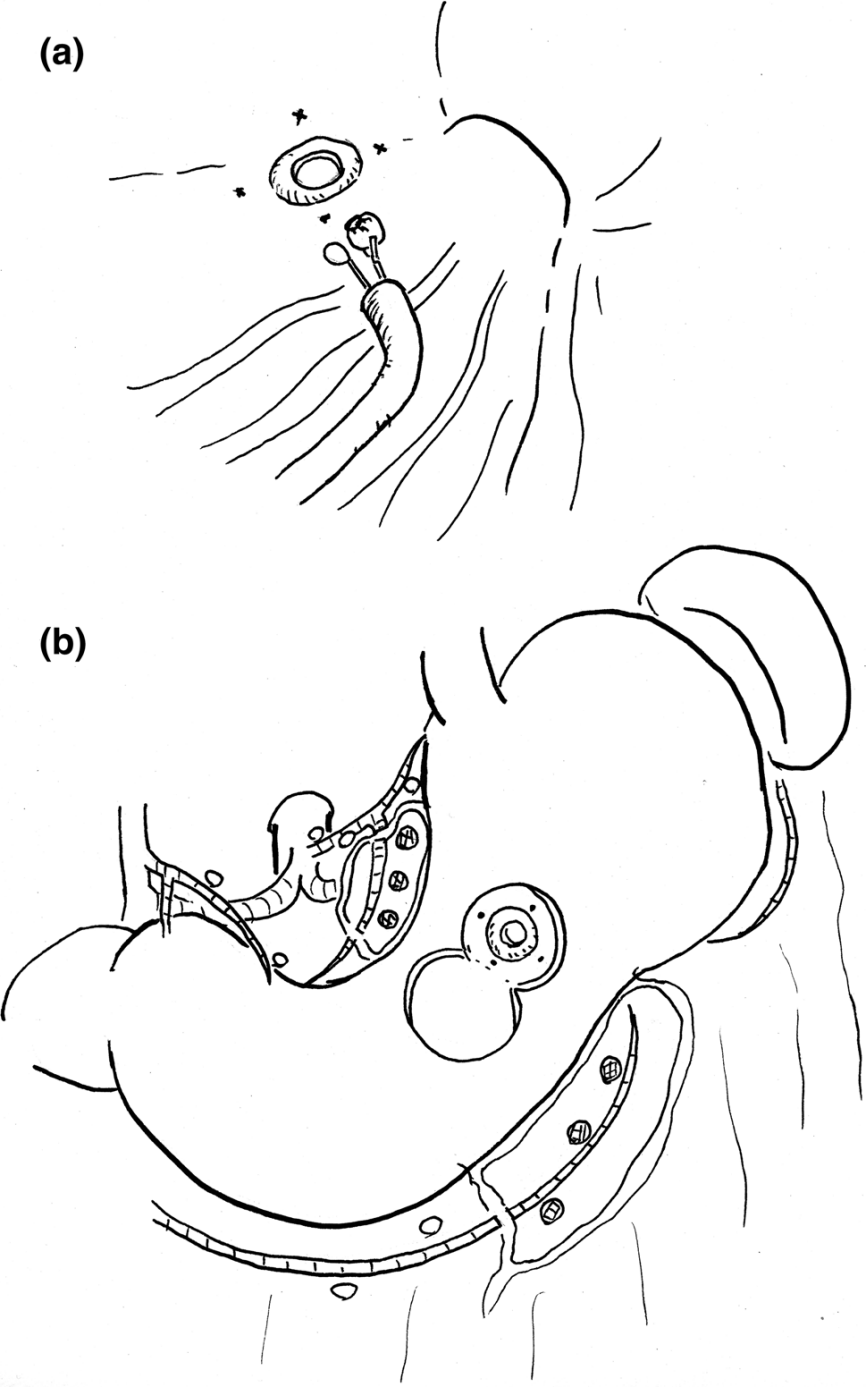
The old technique of local resection for early gastric cancer reported by Dr. Takagi. **a** Before the surgery, tattooing and targeted biopsies are performed at four points around the tumor to ensure a safe margin is achieved, and the center of the main lesion is then resected by EMR to determine the depth of invasion. If the biopsy findings suggest mucosal cancer, then a laparotomy is performed. **b** Local resection of the stomach is performed with the dissection line set beyond the tattooed sites. The sampling dissection of the perigastric nodes dyed by India ink is also performed to assess the lymph node status and staging

**Fig. 5:**
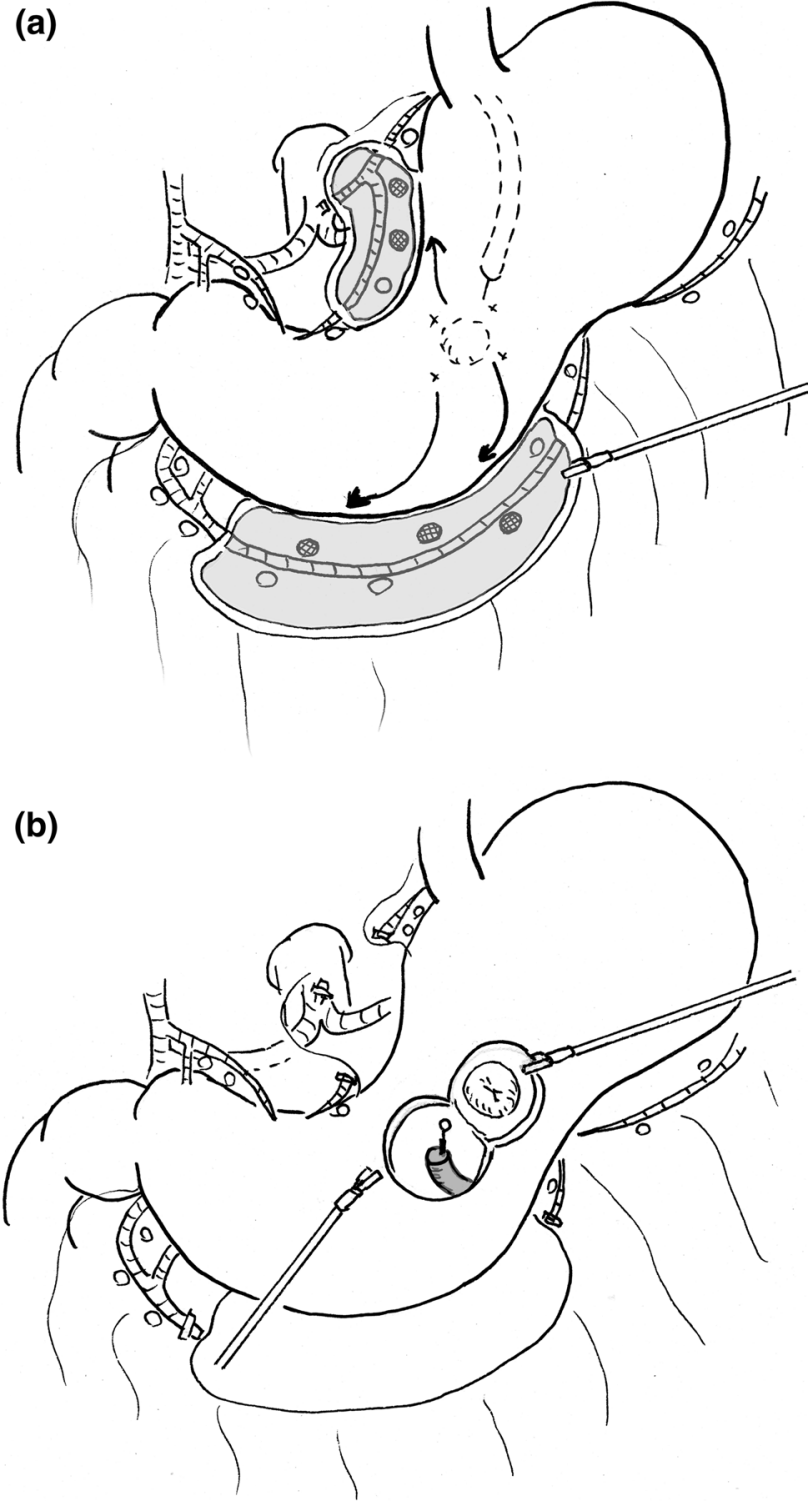
The ideal model for local resection of the stomach for gastric cancer. **a** A laparoscopic fluorescence-guided sentinel node biopsy is performed during surgery to verify a negative lymph node status. The en bloc dissection of the lymphatic basin is performed laparoscopically, and the bright nodes are sent to the pathological section to carry out an intraoperative molecular diagnosis of micrometastasis. **b** The combination of endoscopic full-thickness resection of the stomach using an ESD device and hand suturing closure using a laparoscope or a surgical robot is performed to achieve oncological safety and well preserved functions

Takagi et al. [51] reported a method of local resection combining surgery with EMR for early gastric cancer (Fig. 4) before the era of laparoscopic surgery. This method is surprisingly similar to the method (Fig. 5) of “combining sentinel node biopsy to confirm that the patient is negative for metastasis, performing local resection of the stomach by LECS or EFTR, and repairing it by surgical suturing”, which is the current state-of-the-art investigative therapy. The method by Takagi et al. was then not popularized, but it is likely to be resurrected after 25 years owing to the technical developments of sentinel node biopsy and recent advances in endoscopic surgical equipment.

However, the issues associated with local resection for gastric cancer, other than the indications, have not so far been treated as critically important. The problems of gastric deformation, functional impairment, and multiple gastric cancers are yet to be resolved. Laparoscopic resections are variable, and have not been proven to be superior to routine surgery. In view of these issues, there is thus a tendency to regard local resection of the stomach as an investigative treatment, to be applied only in carefully selected cases.
